# Interpretable machine learning and mendelian randomization identify risk factors for lower extremity arterial embolism and thrombosis

**DOI:** 10.3389/fcvm.2026.1827210

**Published:** 2026-07-24

**Authors:** Xiaodong Li, Guohao Wei, Rui Liu, Qiulin Jiang, Yarong Ma, Xiaolei Sun

**Affiliations:** 1 Department of Interventional Medicine, The Affiliated Hospital of Southwest Medical University, Luzhou, China; 2 Department of Radiology, The Affiliated Hospital of Southwest Medical University, Luzhou, China; 3 The Fourth People’s Hospital of Sichuan Province, West China Chunxi Hospital of Sichuan University, Chengdu, China; 4 Department of General Surgery (Vascular Surgery), The Affiliated Hospital of Southwest Medical University, Luzhou, China; 5 Laboratory of Nucleic Acids in Medicine for National High-Level Talent, Nucleic Acid Medicine of Luzhou Key Laboratory, Southwest Medical University, Luzhou, China; 6 Key Laboratory of Medical Electrophysiology, Ministry of Education & Medical Electrophysiological Key Laboratory of Sichuan Province, Collaborative Innovation Center for Prevention and Treatment of Cardiovascular Disease of Sichuan Province, Institute of Cardiovascular Research, Southwest Medical University, Luzhou, China; 7 Cardiovascular and Metabolic Diseases Key Laboratory of Luzhou, Southwest Medical University, Luzhou, China

**Keywords:** arterial embolism, arterial thrombosis, machine learning, mean platelet volume, mendelian randomization

## Abstract

**Background:**

Lower extremity arterial embolism and thrombosis lead to significant morbidity, but their risk factors are not fully characterized.

**Objectives:**

To identify risk factors and develop an interpretable machine learning (ML) model for predicting lower extremity arterial embolism and thrombosis, with validation using mendelian randomization (MR).

**Methods:**

In this retrospective case-control study, data were collected from patients with lower extremity arterial embolism and thrombosis treated at our department of vascular surgery between January 2018 and November 2025. Predictors were selected using LASSO regression, and 11 ML models were developed using the selected variables. The optimal model was interpreted and implemented as a web-based prediction tool. Clinical utility and model calibration were assessed using decision curve analysis and calibration curves. Key predictors were further assessed using MR and multivariable logistic regression.

**Results:**

XGBoost achieved the highest discrimination, with an AUC of 0.951 (95% CI 0.925–0.972) in the test set. MR analyses indicated that genetically predicted cerebrovascular disease (CD) (OR 1.773; 95% CI 1.043–3.015; *P* = 0.035) and gamma-glutamyl transferase (GGT) (OR 1.327; 95% CI 1.105–1.595; *P* = 0.003) were risk factors, whereas mean platelet volume (MPV) was protective (OR 0.879; 95% CI 0.778–0.993; *P* = 0.038). Multivariable logistic regression confirmed CD (OR 10.19; 95% CI 4.36–23.82; *P* < 0.001) and MPV (OR 0.63; 95% CI 0.52–0.76; *P* < 0.001) as independent predictors.

**Conclusions:**

Interpretable ML combined with MR identified a history of CD and lower MPV as factors associated with risk of lower extremity arterial embolism and thrombosis.

## 1 Introduction

Lower extremity arterial embolism and thrombosis are major causes of acute limb ischemia (ALI). In the United States, the annual incidence of ALI is estimated at 15 to 26 cases per 100,000 persons per year (1), and the burden is increasing with population ageing. Although multiple revascularization options are available—including catheter-directed thrombolysis, mechanical thrombectomy, and surgical embolectomy—clinical outcomes remain unsatisfactory. U.S. data suggest that 1-year mortality and major amputation rates after intervention remain as high as 17% and 34%, respectively (2), attributable in part to delays in diagnosis and treatment (3). Early risk stratification is therefore essential for prevention, timely diagnosis, and optimized management (4); however, a validated clinical prediction model for early detection is still lacking.

Machine learning (ML) can capture nonlinear relationships and interaction effects from high-dimensional, complex data (5, 6); however, its black-box decision process can limit clinical adoption (7). SHapley Additive exPlanations (SHAP) quantify each feature's contribution to an individual prediction using a game-theoretic framework (8, 9), whereas local interpretable model-agnostic explanations (LIME) provide local explanations that are model-agnostic (10, 11). Together, these approaches improve model transparency and may increase clinician trust in ML-based decision support.

Mendelian randomization (MR) leverages genetic variants [single-nucleotide polymorphisms (SNPs)] as instrumental variables to estimate causal effects. Because these instruments are fixed at conception and are generally not influenced by postnatal environmental or behavioral factors, MR can reduce confounding and mitigate reverse causation, thereby strengthening causal inference in observational studies (12).

This study aimed to identify risk factors for lower extremity arterial embolism and thrombosis and to develop and validate an interpretable ML model for early, accurate risk prediction. SHAP and LIME were employed to elucidate feature importance and explain model predictions, with causal relationships further validated using MR and multivariable logistic regression.

## 2 Materials and methods

### 2.1 Study cohort

The study was approved by the Ethics Committee of the Affiliated Hospital of Southwest Medical University (*N*o. KY2025688) in accordance with the Declaration of Helsinki. This study consecutively enrolled patients with lower extremity arterial embolism or thrombosis who underwent surgical treatment in the Department of Vascular Surgery between January 2018 and November 2025. The inclusion criteria were: (1) confirmed clinical diagnosis of lower extremity arterial embolism or thrombosis, established based on surgical findings, preoperative imaging, and clinical presentation; (2) receipt of surgical intervention (open or endovascular). Exclusion criteria included: (1) lower extremity diseases due to other causes (e.g., thromboangiitis obliterans, traumatic thrombosis) (*n* = 70); (2) iatrogenic thrombosis following revascularization procedures (*n* = 55); (3) missing key clinical or laboratory data (*n* = 49). Patients with uncomplicated varicose veins (without concomitant peripheral arterial disease) who were treated in the vascular surgery department of our hospital between January 2018 and November 2025 were recruited as controls. Given the practical difficulty of obtaining comprehensive clinical and laboratory data across various age groups from completely healthy individuals in a hospital-based setting, these patients were selected because they routinely undergo lower extremity vascular ultrasound during preoperative evaluation in our department, which helps exclude patients with concurrent peripheral arterial disease and ensures a reliable negative control status. After applying the predefined inclusion and exclusion criteria, all eligible patients within the predefined timeframe were included in the analysis, yielding a final study cohort of 238 patients with lower extremity arterial embolism and thrombosis and 753 control patients. The patient selection flowchart is presented in Supplementary Figure S1.

### 2.2 Clinical features and data processing

The data collected in this study were sourced from patients' inpatient electronic medical records (EMRs), including basic patient information, history of smoking and alcohol consumption, medical history, and laboratory parameters (first laboratory tests after admission, obtained prior to the administration of any anticoagulants or surgical/interventional procedures). Basic patient information includes age and sex. Medical history includes hypertension (HBP), diabetes mellitus (DM), cerebrovascular disease (CD), and coronary heart disease (CHD). Laboratory parameters included total cholesterol (TC, 2.9–5.18 mmol/L), triglycerides (TG, 0.4–1.7 mmol/L), high-density lipoprotein cholesterol (HDL-C, 1.04–2.08 mmol/L), low-density lipoprotein cholesterol (LDL-C, 1–3.37 mmol/L), apolipoprotein A1 (APOA1, 1.1–1.7 g/L), apolipoprotein B (APOB, 0.8–1.55 g/L), alanine aminotransferase (ALT, 9–50 U/L), aspartate aminotransferase (AST, 15–40 U/L), total protein (TP, 65–85 g/L), albumin (ALB, 40–55 g/L), gamma-glutamyl transferase (GGT, 10–60 U/L), urea (3.2–8.2 mmol/L), uric acid (UA, 208–428 μmol/L), creatinine (Crea, 57–111 μmol/L), glomerular filtration rate (GFR, 75–145 mL/min), fibrinogen (Fib, 2–4 g/L), prothrombin time (PT, 11.0–14.5 s), thrombin time (TT, 14–21 s), activated partial thromboplastin time (APTT, 26–40 s), mean platelet volume (MPV, 9.4–12.5 fl), and platelet count (PLT, 125–350 × 10^9^/L). Cases with any missing variables from the aforementioned list were excluded. Given the relatively small proportion of missing data, a complete-case analysis was adopted. Therefore, only patients with complete information for all study variables were included in the final analysis to preserve the integrity of the original dataset and avoid additional assumptions associated with data imputation. Outliers, defined as values exceeding 1.5 times the interquartile range above the upper quartile or below the lower quartile, were manually reviewed to rule out data entry errors. For patients with multiple records, only the first episode was retained for analysis. The disease and control cohorts were combined and then randomly split into a training set and a test set at a ratio of 7:3.

### 2.3 Selection of variables

Variable selection was performed using least absolute shrinkage and selection operator (LASSO) regression, which imposes an L1 penalty on regression coefficients, shrinking those of non-informative variables toward zero and thus improving model sparsity and stability. The optimal regularization parameter *λ* was chosen by 10-fold cross-validation with the glmnet package (version 4.1-8), using the value of *λ* within one standard error of the minimum cross-validation error.

### 2.4 Model development

Eleven ML models were developed using the LASSO-selected variables, including support vector machine (SVM), neural network, multilayer perceptron (MLP), Gaussian process, gradient boosting machine (GBM), logistic regression, naive Bayes, adaptive boosting (AdaBoost), extreme gradient boosting (XGBoost), random forest, and k-nearest neighbors (KNN). All models were implemented in R using the caret package (version 7.0-1) with 5-fold cross-validation and hyperparameter tuning**.**

### 2.5 Model evaluation

Model performance was evaluated using standard discrimination and accuracy metrics, including the area under the receiver operating characteristic curve (AUC), sensitivity, specificity, positive predictive value (PPV), negative predictive value (NPV), accuracy, F1-score, and Brier score. Calibration curves were further employed to visualize the agreement between predicted and observed risks. Decision curve analysis (DCA) was applied to evaluate the net clinical benefit of the model across a range of threshold probabilities. Calibration curves were generated using the predtools package (version 0.0.3), and DCA was implemented in R using a custom function.

### 2.6 Model explanation

To address the “black-box” nature of ML models and enhance their clinical acceptability, we employed both SHAP and LIME for *post hoc* model interpretation. SHAP, grounded in cooperative game theory, provides both global feature importance and local explanations for individual predictions. LIME was used as a complementary approach to further improve local interpretability. SHAP values were computed using the fastshap package (version 0.1.1) and visualized with the shapviz package (version 0.10.2), whereas LIME analyses were performed with the lime package (version 0.5.3).

### 2.7 Network calculator

The final prediction model was deployed as an R Shiny web application hosted on shinyapps.io (https://www.shinyapps.io), enabling individualized estimation of the risk of lower extremity arterial embolism or thrombosis.

### 2.8 MR analysis

MR analysis is based on three core assumptions: (1) strong association between genetic instruments and the exposure; (2) no direct association with the outcome; and (3) independence from confounders (13). Instrumental variables were selected using a genome-wide significance threshold (*P* < 5 × 10⁻^8^). Linkage disequilibrium was controlled for (clumping distance = 10,000 kb, *r*^2^ < 0.001). The instrument strength was assessed using the F-statistic, calculated as F = [R^2^/(1 − R^2^)] × (N − 2), where R^2^ = 2 × EAF × (1 − EAF) × *β*^2^. Variants with F < 10, indicating weak instruments, were excluded (14). MR analyses were primarily performed using the inverse variance weighted (IVW) method, with a *P* value < 0.05 indicating significant evidence for a causal effect. Sensitivity analyses were conducted using MR Egger and weighted median methods. The robustness of the findings was further assessed by evaluating heterogeneity (Cochran's *Q* test) and horizontal pleiotropy (MR-Egger intercept test). All analyses were implemented with the “TwoSampleMR” R package (version 0.6.8). Genome-Wide Association Study (GWAS) summary data for lower extremity arterial embolism and thrombosis (1,297 cases; 463,106 controls) were sourced from the FinnGen R12. Data for CD, smoking, DM, MPV, AST, urea, GFR, and GGT were obtained from the IEU OpenGWAS and GWAS Catalog. CHD was excluded from MR analysis as it provided no predictive contribution in the model. Complete GWAS data are detailed in Supplementary Materials Table S1, with beta coefficients for CD derived from log (OR).

### 2.9 Statistics

All analyses were conducted using R (version 4.4.2). Continuous variables were expressed as mean ± standard deviation (SD) or median (interquartile range, IQR). For comparisons between two groups, the student's t test was used for normally distributed data, and the Mann–Whitney U test was used for non-normally distributed data. The normality of distribution was assessed using the Shapiro–Wilk test. Categorical variables were presented as *n* (%) and compared by chi-square tests. Dose-response relationships were assessed using Restricted Cubic Spline (RCS). Statistical significance was set at a two-sided *P* value < 0.05; *P* values ≥ 0.001 were reported as exact values, whereas *P* values < 0.001 were reported as *P* < 0.001. Multivariable logistic regression was used to assess independent risk factors, with female sex as the reference category for gender and “no” as the reference for all other categorical variables. Key predictors were identified as the intersection of variables demonstrating significant associations in both multivariable logistic regression and MR analysis.

## 3 Results

### 3.1 Baseline clinical information

The disease group (lower extremity arterial embolism and thrombosis) comprised 238 patients, while the control group (patients with simple varicose veins) included 753 patients. In the analysis of lower extremity arterial embolism, the training set contained 695 samples and the test set contained 296 samples. Compared with the control group, the disease group had a higher proportion of males (66% vs. 48%), was older (76 vs. 60 years), and demonstrated a higher prevalence of diabetes (24% vs. 6%), hypertension (48% vs. 22%), cerebrovascular disease (23% vs. 3%), and coronary heart disease (17% vs. 2%). Additionally, the disease group contained more patients with a history of smoking (37% vs. 21%) and alcohol consumption (33% vs. 21%). Regarding lipid profiles, the disease group showed lower levels of TC (4.51 vs. 4.67 mmol/L), LDL-C (2.68 vs. 2.82 mmol/L), and ApoA1 (1.39 vs. 1.47 g/L). Patients in the disease group showed impaired hepatic and renal function, as evidenced by elevated levels of ALT (22.95 vs. 18.80 U/L), AST (26.35 vs. 21.00 U/L), GGT (34.35 vs. 20.00 U/L), TP (68.65 vs. 67.50 g/L), urea (6.52 vs. 5.76 mmol/L), UA (339.60 vs. 327.60 *μ*mol/L), and Crea (75.85 vs. 64.00 *μ*mol/L), along with decreased ALB (40.20 vs. 40.80 g/L) and GFR (82.15 vs. 98.10 mL/min). In terms of coagulation function, the disease group exhibited prolonged PT (13.30 vs. 12.90 s), shortened APTT (32.90 vs. 34.70 s) and TT (17.40 vs. 18.00 s), and elevated Fib (3.86 vs. 2.81 g/L). Meanwhile, MPV was lower in the disease group (9.80 vs. 10.80 fl). Variables were generally well-balanced between the training and test sets, except for slightly older age in the training set (64 vs. 61 years) (Table 1).

**Table 1:** Baseline characteristics.

			By group			By dataset		
Variables	Reference range (Unit)	Overall *N* = 991^a^	Control *N* = 753^a^	Disease *N* = 238^a^	*p* ^b^	Training *N* = 695^a^	Test *N* = 296^a^	*p* ^b^
Sex	—				<0.001			0.812
Female		468 (47%)	388 (52%)	80 (34%)		326 (47%)	142 (48%)	
Male		523 (53%)	365 (48%)	158 (66%)		369 (53%)	154 (52%)	
year	year	63 (55, 73)	60 (54, 69)	76 (66, 83)	<0.001	64 (56, 74)	61 (54, 72)	0.050
DM	—				<0.001			0.653
No		889 (90%)	707 (94%)	182 (76%)		621 (89%)	268 (91%)	
Yes		102 (10%)	46 (6%)	56 (24%)		74 (11%)	28 (9%)	
HBP	—				<0.001			0.123
No		708 (71%)	584 (78%)	124 (52%)		486 (70%)	222 (75%)	
Yes		283 (29%)	169 (22%)	114 (48%)		209 (30%)	74 (25%)	
CD	—				<0.001			0.139
No		917 (93%)	734 (97%)	183 (77%)		637 (92%)	280 (95%)	
Yes		74 (7%)	19 (3%)	55 (23%)		58 (8%)	16 (5%)	
CHD	—				<0.001			0.910
No		937 (95%)	740 (98%)	197 (83%)		658 (95%)	279 (94%)	
Yes		54 (5%)	13 (2%)	41 (17%)		37 (5%)	17 (6%)	
Smoking	—				<0.001			0.599
No		744 (75%)	594 (79%)	150 (63%)		518 (75%)	226 (76%)	
Yes		247 (25%)	159 (21%)	88 (37%)		177 (25%)	70 (24%)	
Alcohol consumption	—				<0.001			0.670
No		757 (76%)	597 (79%)	160 (67%)		534 (77%)	223 (75%)	
Yes		234 (24%)	156 (21%)	78 (33%)		161 (23%)	73 (25%)	
TC	2.9–5.18 (mmol/L)	4.64 (4.04, 5.28)	4.67 (4.09, 5.28)	4.51 (3.80, 5.22)	0.040	4.63 (4.03, 5.25)	4.68 (4.05, 5.30)	0.655
TG	0.4–1.7 (mmol/L)	1.27 (0.93, 1.82)	1.27 (0.92, 1.76)	1.27 (0.96, 1.89)	0.394	1.28 (0.93, 1.84)	1.25 (0.93, 1.73)	0.563
HDL-C	1.04–2.08 (mmol/L)	1.34 (1.11, 1.59)	1.35 (1.13, 1.57)	1.28 (1.06, 1.63)	0.494	1.35 (1.12, 1.60)	1.34 (1.11, 1.57)	0.630
LDL-C	1–3.37 (mmol/L)	2.80 (2.26, 3.28)	2.82 (2.33, 3.30)	2.68 (2.02, 3.27)	0.010	2.78 (2.26, 3.27)	2.82 (2.28, 3.31)	0.442
ApoA1	1.1–1.7 (g/L)	1.45 (1.29, 1.62)	1.47 (1.31, 1.62)	1.39 (1.20, 1.63)	<0.001	1.45 (1.29, 1.62)	1.44 (1.28, 1.62)	0.876
ApoB	0.8–1.55 (g/L)	0.88 (0.72, 1.03)	0.88 (0.72, 1.02)	0.91 (0.72, 1.07)	0.074	0.88 (0.73, 1.03)	0.88 (0.71, 1.05)	0.849
ALT	9–50 (U/L)	19.20 (14.10, 27.60)	18.80 (14.00, 25.70)	22.95 (14.70, 36.20)	<0.001	19.30 (14.30, 27.60)	18.85 (13.55, 27.40)	0.301
AST	15–40 (U/L)	21.70 (18.10, 27.20)	21.00 (17.90, 25.30)	26.35 (19.60, 45.30)	<0.001	21.70 (18.30, 27.70)	21.80 (17.85, 26.35)	0.620
TP	65–85 (g/L)	67.70 (63.90, 71.80)	67.50 (63.70, 71.30)	68.65 (64.10, 73.30)	0.052	67.30 (63.40, 71.60)	68.05 (64.45, 72.10)	0.182
ALB	40–55 (g/L)	40.70 (38.50, 43.20)	40.80 (38.70, 43.20)	40.20 (37.60, 42.90)	0.005	40.70 (38.40, 43.20)	40.70 (38.60, 43.20)	0.527
GGT	10–60 (U/L)	22.00 (15.40, 37.60)	20.00 (14.00, 30.40)	34.35 (22.70, 57.70)	<0.001	22.50 (15.70, 38.10)	21.40 (14.90, 36.40)	0.595
Urea	3.2–8.2 (mmol/L)	5.88 (4.88, 7.07)	5.76 (4.80, 6.78)	6.52 (5.19, 9.40)	<0.001	5.84 (4.80, 7.11)	5.95 (5.02, 6.94)	0.448
UA	208–428 (*μ*mol/L)	330.60 (277.00, 388.80)	327.60 (278.70, 383.00)	339.60 (268.30, 427.00)	0.049	329.00 (270.50, 386.40)	336.50 (285.30, 395.10)	0.060
Crea	57–111 (μmol/L)	66.00 (56.70, 77.90)	64.00 (55.20, 73.30)	75.85 (61.30, 92.70)	<0.001	66.00 (57.00, 77.70)	66.10 (56.10, 78.40)	0.866
GFR	75–145 (mL/min)	95.20 (84.00, 104.40)	98.10 (90.10, 105.70)	82.15 (61.00, 92.70)	<0.001	94.50 (83.50, 103.90)	96.55 (84.85, 105.35)	0.256
PT	11.0–14.5 (s)	12.90 (12.50, 13.50)	12.90 (12.50, 13.30)	13.30 (12.50, 14.10)	<0.001	12.90 (12.50, 13.40)	13.00 (12.40, 13.60)	0.752
APTT	26–40 (s)	34.40 (31.40, 37.30)	34.70 (32.10, 37.30)	32.90 (29.10, 37.80)	<0.001	34.40 (31.30, 37.40)	34.30 (31.55, 37.25)	0.738
TT	14–21 (s)	17.90 (17.10, 18.80)	18.00 (17.30, 18.80)	17.40 (16.30, 18.80)	<0.001	17.90 (17.10, 18.70)	18.10 (17.10, 19.10)	0.145
Fib	2–4 (g/L)	2.93 (2.55, 3.47)	2.81 (2.49, 3.20)	3.86 (3.12, 5.04)	<0.001	2.93 (2.57, 3.49)	2.94 (2.54, 3.42)	0.497
MPV	9.4–12.5 (fl)	10.60 (9.70, 11.60)	10.80 (10.00, 11.80)	9.80 (9.00, 10.90)	<0.001	10.70 (9.70, 11.60)	10.50 (9.70, 11.60)	0.640
PLT	125–350 (×10^9^/L)	197.00 (160.00, 241.00)	197.00 (162.00, 234.00)	199.00 (150.00, 271.00)	0.178	197.00 (160.00, 239.00)	197.00 (162.00, 244.50)	0.493

a n (%); mean ± SD or median (Q1, Q3).

b Continuous variables: normally distributed data are presented as mean ± SD and compared using Student's t test; non-normally distributed data are presented as median (IQR) and compared using the Mann–Whitney U test. Categorical variables were compared using the chi-square test.

DM, diabetes mellitus; HBP, hypertension; CD, cerebrovascular disease; CHD, coronary heart disease; TC: total cholesterol; TG, triglycerides; HDL-C, high-density lipoprotein cholesterol; LDL-C, low-density lipoprotein cholesterol; ApoA1, apolipoprotein A1; ApoB, apolipoprotein B; ALT, alanine aminotransferase; AST, aspartate aminotransferase; TP, total protein; ALB, albumin; GGT, gamma-glutamyl transferase; UA, uric acid; Crea, creatinine; GFR, glomerular filtration rate; PT, prothrombin time; APTT, activated partial thromboplastin time; TT, thrombin time; Fib, fibrinogen; MPV, mean platelet volume; PLT, platelet count.

To assess the potential impact of complete-case analysis on selection bias, baseline characteristics were compared between included patients and those excluded because of missing data (Supplementary Materials Table S2). Except for ALT (22.95 vs. 17.00 U/L) and TT (17.40 vs. 16.90 s), no statistically significant differences were observed for most baseline characteristics, including age, sex, major comorbidities, lipid profiles, renal function parameters, and MPV. These findings indicate that the baseline characteristics of the included and excluded patients were highly similar, suggesting a minimal risk of substantial selection bias resulting from the complete-case analysis.

### 3.2 Selection of predictor variables

**Figure 1:**
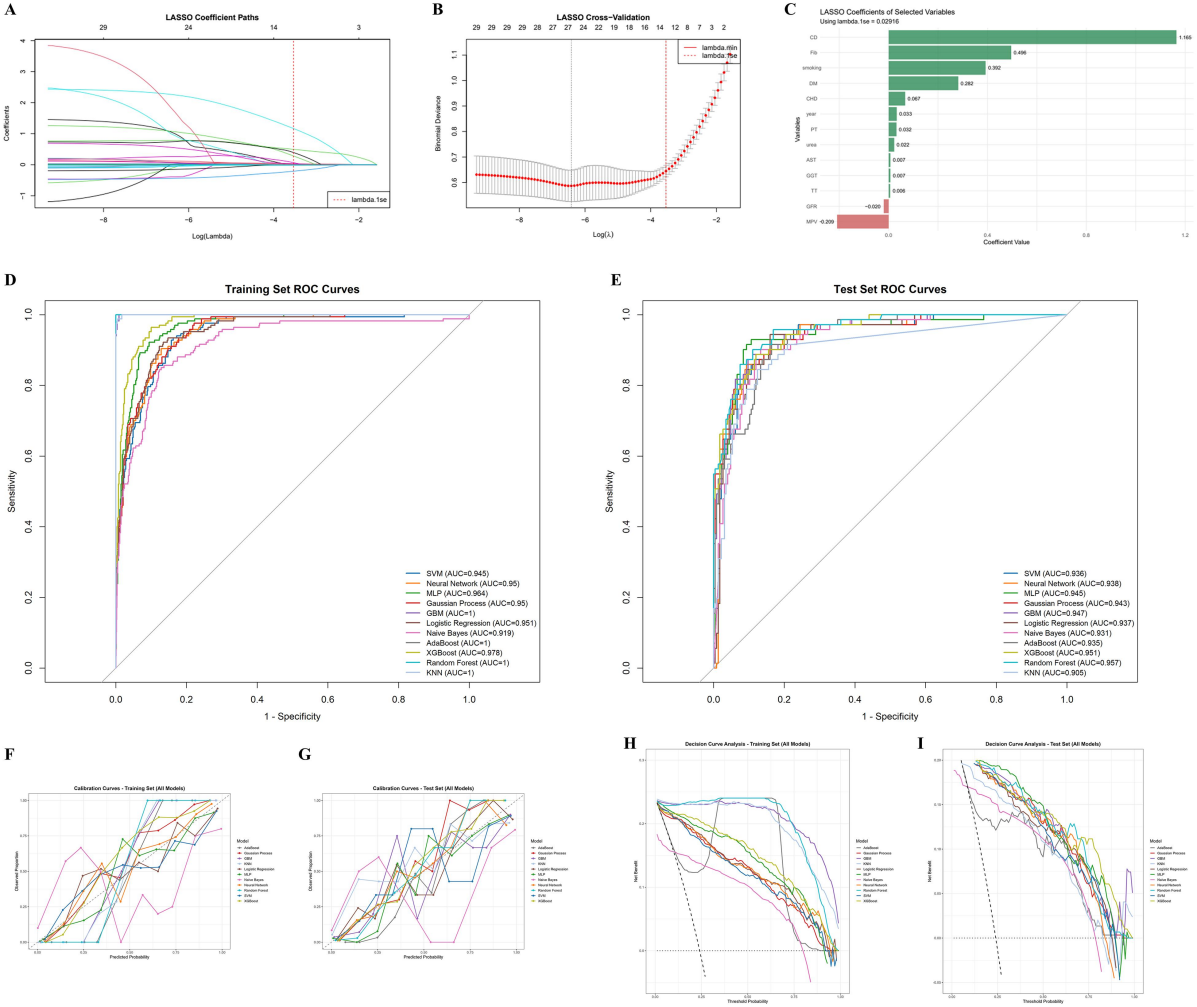
Feature selection, performance evaluation, and clinical utility assessment of machine learning models. **(A)** LASSO coefficient paths; **(B)** LASSO cross-validation; **(C)** LASSO selected variables and coefficients; **(D)** Training set Receiver Operating Characteristic (ROC) curves of all models; **(E)** Test set ROC curves of all models; **(F)** Calibration curves in the training set; **(G)** Calibration curves in the test set; **(H)** Decision curve analysis (DCA) in the training set; **(I)** DCA in the test set. In calibration curves **(F),(G)**, the diagonal line represents perfect prediction; closer alignment indicates better calibration. In DCA **(H)**,**(I)**, the curves show the net benefit across different threshold probabilities, with the horizontal line representing the “treat-none” strategy and the oblique dashed line representing the “treat-all” strategy.

LASSO regression retained 13 predictors with non-zero coefficients for model development, including CD, Fib, smoking, DM, MPV, CHD, year, PT, urea, GFR, AST, GGT, and TT (Figures 1A–C).

### 3.3 Model development, performance comparison and sensitivity analysis

The eleven ML models showed good discriminative performance for predicting lower extremity arterial embolism and thrombosis, with training AUCs ranging from 0.919 to 1.000 and test AUCs ranging from 0.905 to 0.957 (Figures 1D,E). After excluding overfitted models (training AUC = 1), the XGBoost algorithm demonstrated the most favorable balance of discrimination and generalization, achieving a training AUC of 0.978 and a test AUC of 0.951. The model also demonstrated strong performance in accuracy (0.922 training; 0.899 test), specificity (0.975 training; 0.973 test), and precision (0.906 training; 0.887 test), although sensitivity was comparatively lower (0.754 training; 0.662 test). A comprehensive summary of all performance metrics is provided in Table 2. Calibration curves showed that XGBoost followed the ideal diagonal line reasonably well across most probability ranges in both the training (Figure 1F) and test sets (Figure 1G), indicating generally good agreement between predicted and observed risks. DCA in the training (Figure 1H) and test sets (Figure 1I) demonstrated that the XGBoost model yielded a higher net clinical benefit across a wide range of threshold probabilities.

**Table 2:** Detailed performance metrics of all machine learning models in the training and test sets.

Model	Dataset	AUC (95% CI)	Accuracy	Sensitivity	Specificity	PPV	NPV	F1
SVM	Training	0.945 (0.927–0.961)	0.879	0.695	0.938	0.779	0.907	0.734
SVM	Test	0.936 (0.901–0.964)	0.885	0.732	0.933	0.776	0.917	0.754
Neural Network	Training	0.95 (0.933–0.964)	0.891	0.689	0.955	0.827	0.906	0.752
Neural Network	Test	0.938 (0.908–0.965)	0.892	0.732	0.942	0.8	0.918	0.765
MLP	Training	0.964 (0.951–0.976)	0.915	0.844	0.938	0.81	0.95	0.827
MLP	Test	0.945 (0.912–0.973)	0.909	0.831	0.933	0.797	0.946	0.814
Gaussian Process	Training	0.950 (0.933–0.965)	0.892	0.653	0.968	0.865	0.898	0.744
Gaussian Process	Test	0.943 (0.912–0.970)	0.889	0.676	0.956	0.828	0.903	0.744
GBM	Training	1.000 (0.999–1.000)	0.99	0.97	0.996	0.988	0.991	0.979
GBM	Test	0.947 (0.916–0.969)	0.902	0.817	0.929	0.784	0.941	0.8
Logistic Regression	Training	0.951 (0.934–0.965)	0.888	0.683	0.953	0.82	0.905	0.745
Logistic Regression	Test	0.937 (0.904–0.963)	0.885	0.732	0.933	0.776	0.917	0.754
Naive Bayes	Training	0.919 (0.893–0.943)	0.862	0.635	0.934	0.752	0.89	0.688
Naive Bayes	Test	0.931 (0.900–0.959)	0.872	0.676	0.933	0.762	0.901	0.716
AdaBoost	Training	1.000 (1.000–1.000)	1.000	1.000	1.000	1.000	1.000	1.000
AdaBoost	Test	0.935 (0.904–0.961)	0.851	0.662	0.911	0.701	0.895	0.681
XGBoost	Training	0.978 (0.970–0.986)	0.922	0.754	0.975	0.906	0.926	0.824
XGBoost	Test	0.951 (0.925–0.972)	0.899	0.662	0.973	0.887	0.901	0.758
Random Forest	Training	1.000 (1.000–1.000)	1.000	1.000	1.000	1.000	1.000	1.000
Random Forest	Test	0.957 (0.932–0.977)	0.902	0.704	0.964	0.862	0.912	0.775
KNN	Training	1.000 (0.999–1.000)	0.991	0.976	0.996	0.988	0.992	0.982
KNN	Test	0.905 (0.860–0.946)	0.868	0.62	0.947	0.786	0.887	0.693

SVM, support vector machine; MLP, multilayer perceptron; GBM, gradient boosting machine; AdaBoost, adaptive boosting; XGBoost, extreme gradient boosting; KNN, k-nearest neighbors; AUC, area under the curve; 95% CI: 95% confidence interval; PPV, positive predictive value; NPV, negative predictive value; F1, F1-score.

### 3.4 Model explanation

**Figure 2:**
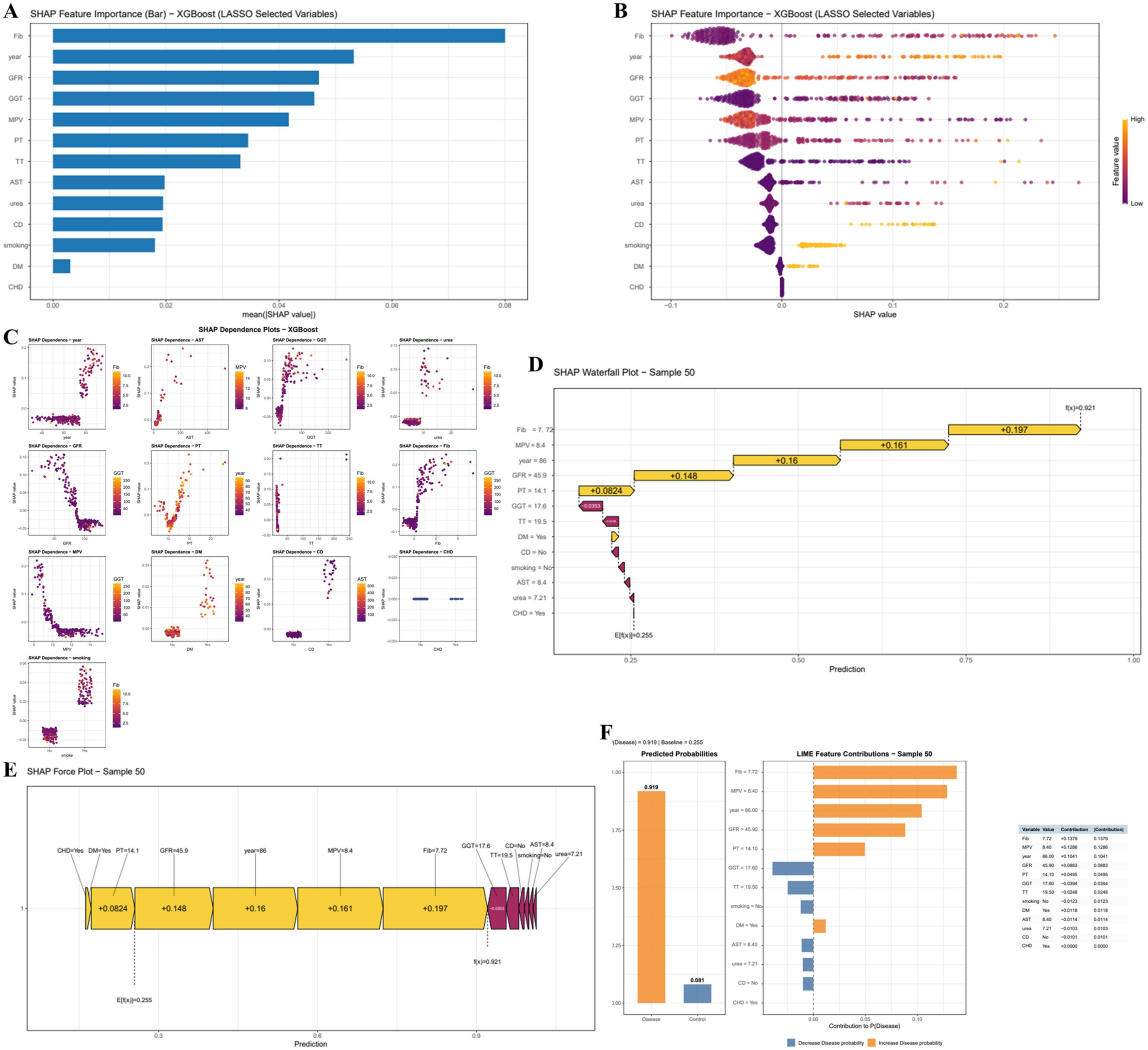
Machine learning (ML) interpretability analysis results. **(A)** Feature importance ranked by mean absolute SHapley Additive exPlanations (SHAP) values; **(B)** SHAP beeswarm plot of SHAP value distributions for variables included in the model, points to the right indicate higher predicted risk, and color reflects feature value (orange high, purple low); **(C)** SHAP dependence plots for variables included in the model, illustrating feature–prediction relationships; **(D)** Waterfall plot for high risk sample 50; **(E)** Force plot for high risk sample 50; **(F)** LIME results for high-risk sample 50, arranged right to left as predicted-probability bar chart, feature-contribution plot, and feature-value table. In the SHAP waterfall plot, SHAP force plot, and LIME explanation, orange indicates positive contributions that push the model output higher, whereas purple or blue indicates negative contributions that push it lower. Bar length reflects the contribution magnitude (as labeled), and adding the feature contributions to the base value yields the prediction for the instance.

SHAP analysis of the XGBoost model, developed using LASSO-selected features, revealed the order of feature importance based on mean absolute SHAP values as Fib, year, GFR, GGT, MPV, PT, TT, AST, CD, urea, smoking, and DM, while CHD exhibited a SHAP value of zero (Figure 2A). The SHAP beeswarm plot illustrates the directional impact of feature variations on model predictions, where each point represents an individual sample. In the XGBoost model, advanced age, elevated Fib, GGT, PT, TT, AST, and urea levels, decreased GFR and MPV levels, and positive history of CD, DM, or smoking were identified as predictive factors for lower extremity arterial embolism and thrombosis. CHD demonstrated no predictive influence (Figure 2B).

SHAP dependence plots further delineated the specific relationship patterns between individual features and the risk of lower extremity arterial embolism and thrombosis. These plots illustrate how changes in each continuous variable affect the predicted probability, while for categorical variables, they reveal which category elevates risk. For instance, a history of DM significantly increased the predicted probability of embolism and thrombosis (Figure 2C).

SHAP waterfall plots (Figure 2D) and force plots (Figure 2E) enabled model interpretation at the individual level. The predicted probability of embolism and thrombosis reached 0.919 in high-risk Sample 50 with lower extremity arterial embolism and thrombosis, with the most significant positive contributions from elevated fib (7.72, +0.197), decreased MPV (8.4, +0.1286), advanced age (86 years, +0.1041), reduced GFR (45.9, +0.0883), and prolonged PT (14.1, +0.0495), along with additional positive effects from specific levels of GGT, urea and AST, while negative contributions came from the absence of CD and smoking history, a history of DM showed minimal positive effect, and CHD history demonstrated no predictive value. In the visual representations, orange indicates positive contributions and purple indicates negative contributions. LIME analysis provided additional interpretability at the individual level. For high-risk sample 50 (Figure 2F), the predicted probability of lower extremity arterial embolism and thrombosis reached 95%, with elevated Fib and AST, decreased MPV and GFR, advanced age, and CD history identified as key predictors.

### 3.5 MR and multivariable regression analyses

**Figure 3:**
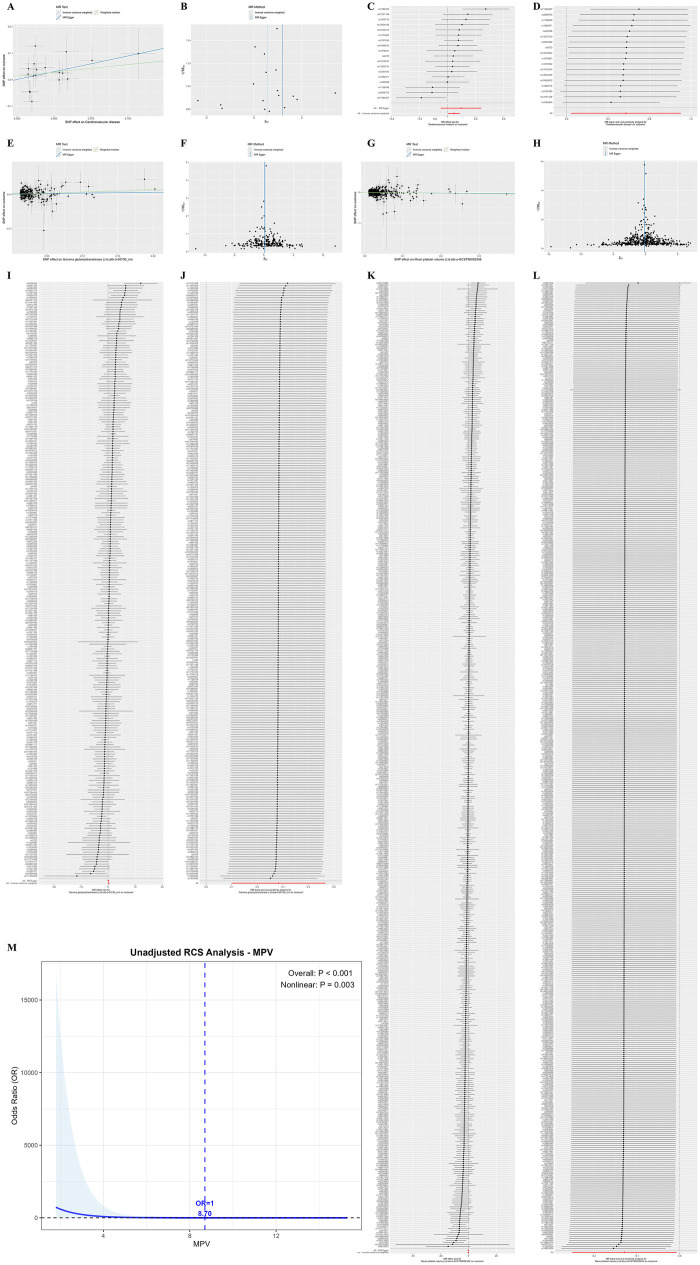
Mendelian randomization (MR) analysis results for cerebrovascular disease (CD), gamma-glutamyl transferase (GGT), and mean platelet volume (MPV) and restricted cubic spline (RCS) results for MPV. **(A)** Scatter plot for CD; **(B)** Funnel plot for CD; **(C)** Forest plot for CD; **(D)** Leave-one-out sensitivity analysis for CD; **(E)** Scatter plot for GGT; **(F)** Funnel plot for GGT; **(G)** Scatter plot for MPV; **(H)** Funnel plot for MPV; **(I)** Forest plot for GGT; **(J)** Leave-one-out sensitivity analysis for GGT; **(K)** Forest plot for MPV; **(L)** Leave-one-out sensitivity analysis for MPV; **(M)** Restricted cubic spline (RCS) analysis for MPV. The MR scatter plots show single nucleotide polymorphism (SNP) specific exposure and outcome effects with the fitted causal estimate. The MR funnel plots summarize the distribution of SNP-specific estimates to evaluate heterogeneity. The MR forest plots present causal estimates across MR methods. The leave-one-out analyses assess the influence of individual SNPs on the overall estimate. The RCS curve shows the association between MPV and thrombotic risk across the MPV range and allows assessment of nonlinearity.

To further identify potential causal risk factors, MR analysis was performed for predictor variables from the model, excluding basic demographic and coagulation parameters, and was supplemented by multivariable logistic regression for validation. MR analyses were conducted for CD, DM, MPV, AST, urea, smoking, GFR, and GGT. The MR results (Supplementary Table S3) provided evidence of positive associations of CD (OR: 1.773, 95% CI: 1.043–3.015, *P* = 0.035) and GGT (OR: 1.327, 95% CI: 1.105–1.595, *P* = 0.003) with lower extremity arterial embolism and thrombosis, with no evidence of horizontal pleiotropy or heterogeneity (*P* > 0.05). MPV showed a negative association with lower extremity arterial embolism and thrombosis (OR: 0.879, 95% CI: 0.778–0.993, *P* = 0.038), with no evidence of horizontal pleiotropy but significant heterogeneity (*P* < 0.001). It should be noted that some degree of heterogeneity is acceptable in MR analyses, particularly when a larger number of SNP instruments are included. No evidence of a causal relationship was found for smoking, DM, urea, GFR, or AST (Supplementary Table S3). Figures 3A–L presents the MR results for the significant variables (CD, GGT, and MPV), including scatter plots, funnel plots, leave-one-out sensitivity analyses, and forest plots. Multivariable logistic regression (Table 3) showed that, among the variables with causal evidence from MR analysis, CD history (OR: 10.19, 95% CI: 4.36–23.82, *P* < 0.001) was positively associated with lower extremity arterial embolism and thrombosis, whereas MPV level (OR: 0.63, 95% CI: 0.52–0.76, *P* < 0.001) showed a negative association. GGT level demonstrated no significant association (OR: 1.00, 95% CI: 1.00–1.00, *P* = 0.843). Integrating the findings from both MR analysis and multivariable logistic regression, CD and MPV were identified as potential causal risk factors for lower extremity arterial embolism and thrombosis. Although GGT showed a significant association in the MR analysis, it did not remain independently associated with the outcome in the multivariable logistic regression model, suggesting that its clinical effect may be influenced by other correlated factors.

**Table 3:** Univariable and multivariable logistic regression analysis.

Variable	Univariable OR (95% CI)	Univariable *P*	Multivariable OR (95% CI)	Multivariable *P*
Sex	2.10 (1.55–2.85)	<0.001	5.40 (2.40–12.11)	<0.001
year	1.11 (1.09–1.13)	<0.001	1.03 (0.99–1.07)	0.115
DM	4.73 (3.10–7.22)	<0.001	2.92 (1.43–5.95)	0.003
HBP	3.18 (2.34–4.32)	<0.001	0.88 (0.49–1.57)	0.661
CD	11.61 (6.72–20.05)	<0.001	10.19 (4.36–23.82)	<0.001
CHD	11.85 (6.23–22.54)	<0.001	2.13 (0.68–6.63)	0.192
Smoking	2.19 (1.60–3.01)	<0.001	1.90 (0.85–4.21)	0.116
Alcohol consumption	1.87 (1.35–2.58)	<0.001	0.79 (0.36–1.73)	0.551
TC	0.89 (0.77–1.04)	0.140	0.72 (0.28–1.85)	0.495
TG	1.03 (0.93–1.13)	0.625	1.18 (0.86–1.62)	0.301
HDL-C	1.01 (0.68–1.50)	0.966	9.66 (1.47–63.64)	0.018
LDL-C	0.82 (0.68–0.99)	0.038	0.21 (0.07–0.60)	0.004
ApoA1	0.36 (0.20–0.65)	<0.001	0.24 (0.02–2.73)	0.248
ApoB	2.01 (1.11–3.64)	0.021	653.95 (40.49–10,562.37)	<0.001
ALT	1.02 (1.01–1.02)	<0.001	0.98 (0.96–1.00)	0.093
AST	1.03 (1.02–1.03)	<0.001	1.03 (1.01–1.05)	<0.001
TP	1.01 (0.99–1.03)	0.365	0.94 (0.88–1.00)	0.045
ALB	0.92 (0.89–0.96)	<0.001	1.11 (1.00–1.24)	0.045
GGT	1.01 (1.00–1.01)	<0.001	1.00 (1.00–1.00)	0.843
urea	1.35 (1.26–1.45)	<0.001	1.15 (1.01–1.31)	0.034
UA	1.00 (1.00–1.00)	<0.001	1.00 (1.00–1.00)	0.880
Crea	1.03 (1.03–1.04)	<0.001	0.97 (0.95–1.00)	0.030
GFR	0.95 (0.94–0.95)	<0.001	0.95 (0.92–0.99)	0.012
PT	1.47 (1.29–1.67)	<0.001	1.68 (1.35–2.09)	<0.001
APTT	1.00 (0.98–1.02)	0.959	0.84 (0.79–0.89)	<0.001
TT	1.03 (1.00–1.07)	0.082	1.18 (1.05–1.34)	0.007
Fib	3.37 (2.77–4.10)	<0.001	3.60 (2.53–5.14)	<0.001
MPV	0.62 (0.55–0.70)	<0.001	0.63 (0.52–0.76)	<0.001
PLT	1.00 (1.00–1.01)	<0.001	1.00 (0.99–1.00)	0.335

DM, diabetes mellitus; HBP, hypertension; CD, cerebrovascular disease; CHD, coronary heart disease; TC, total cholesterol; TG, triglycerides; HDL-C, high-density lipoprotein cholesterol; LDL-C, low-density lipoprotein cholesterol; ApoA1, apolipoprotein A1; ApoB, apolipoprotein B; ALT, alanine aminotransferase; AST, aspartate aminotransferase; TP, total protein; ALB, albumin; GGT, gamma-glutamyl transferase; UA, uric acid; Crea, creatinine; GFR, glomerular filtration rate; PT, prothrombin time; APTT, activated partial thromboplastin time; TT, thrombin time; Fib, fibrinogen; MPV, mean platelet volume; PLT, platelet count; OR, odds ratio; 95% CI, 95% confidence interval.

### 3.6 RCS analysis

To determine the critical threshold at which a continuous variable increases the risk of lower extremity arterial embolism and thrombosis, RCS analysis was performed for MPV. The RCS analysis (Figure 3M) revealed a significant overall association between MPV and thrombotic risk (overall *P* < 0.001), with evidence of nonlinearity (nonlinear *P* = 0.003). The spline curve showed a progressively increased risk with decreasing MPV levels, with a notably higher predicted probability of thrombotic events when MPV fell below 8.70 fl. This association was attenuated after multivariable adjustment, and the evidence for nonlinearity was no longer significant (Supplementary Figure S2).

### 3.7 Web calculator

The prediction model for lower extremity arterial embolism and thrombosis is available online at https://lixiaodong1999.shinyapps.io/thrombosis-risk-predictor/. This web-based platform allows researchers and clinicians to assess patient-specific risk by entering the predictor variables, and it returns both the estimated disease probability and the corresponding SHAP values for feature interpretation. However, it is crucial to emphasize that this tool is currently strictly for exploratory and research purposes. Given the lack of external validation, it should not be used as a standalone basis for direct clinical decision-making until its performance is rigorously validated in independent, multi-center cohorts. The layout of the user interface is illustrated in Supplementary material Figure S3.

## 4 Discussion

By integrating ML and MR analyses with multivariable logistic regression, this study identified a history of CD and lower MPV as independent predictors of lower extremity arterial embolism and thrombosis. RCS analysis further revealed a marked increase in risk when MPV decreased below 8.70 fl.

Beyond conventional coagulation markers, routine hematological parameters have emerged as valuable tools for risk stratification across a broad spectrum of thrombotic disorders. Blood cellular indices have demonstrated prognostic significance in acute pulmonary embolism (15), while baseline hemoglobin levels have been shown to predict clinical outcomes in venous thromboembolism (16). Collectively, these findings suggest that simple, cost-effective, and universally available hematological indices can provide valuable risk stratification across thrombotic disorders. MPV is generally regarded as an indicator of platelet activity, as larger platelets exhibit greater adhesion, aggregation, and procoagulant potential (17, 18). Consistent with this concept, elevated MPV has been associated with increased thrombotic or embolic risk in patients with coronary disease and CD (19–22). In peripheral arterial occlusive disease, however, studies have found an association between lower MPV levels and higher incidence of chronic limb-threatening ischemia (23), and these patients frequently present with thrombotic lumen occlusion (24, 25). Similar findings have also been reported in venous thrombosis. Studies in high-risk groups, including patients with cancer, have suggested that elevated MPV may inversely associate with venous thromboembolism (VTE) (26–28), and two large retrospective cohorts consistently observed lower MPV in patients with deep vein thrombosis (DVT) (29, 30).

Importantly, smaller platelets are not necessarily functionally impaired. Upon adenosine diphosphate (ADP) stimulation, smaller platelets have been shown to exhibit higher *P*-selectin expression (31), which may enhance leukocyte recruitment and stabilize platelet–leukocyte adhesion within thrombi. This effect can be further amplified by procoagulant mediators released from leukocytes, potentially accelerating thrombus propagation. Moreover, under comparable extracellular calcium concentrations, the reactivity of small and large platelets does not differ significantly (32), suggesting that small platelets retain full capacity to participate in thrombus formation. In addition, reduced platelet volume may be accompanied by increased platelet-derived microparticles with enhanced surface reactivity. Through high cross-reactivity with tissue factor (TF) and platelet aggregation receptors, smaller platelets ultimately promote arterial thrombus formation (33). The role of MPV in arterial embolism remains controversial. Although most studies report a positive association between MPV and thromboembolism, some evidence suggests that MPV is not associated with left atrial thrombus formation in patients with atrial fibrillation (34), implying a limited impact on embolic risk in this setting.

Clinically, our analysis identified a history of CD as an important risk factor for lower extremity arterial embolism or thrombosis. This association may reflect shared risk characteristics, including advanced age and metabolic abnormalities. In addition, CD can indicate systemic high-risk pathophysiology, such as advanced atherosclerosis or atrial fibrillation, which may also predispose to thrombotic events in the lower extremity arteries (35, 36). Existing evidence suggests that a history of CD, particularly stroke, is associated with an increased risk of peripheral arterial embolism (37). A higher prevalence of prior CD has also been reported among patients with ALI (38, 39). In patients with ALI, CD history has been identified as an independent risk factor for in-hospital complications (38). Mechanistically, elevated levels of neutrophil extracellular traps (NETs) have been observed in CD (40–43), and NETs may potentiate coagulation by interacting with coagulation factors and facilitating platelet–neutrophil aggregate formation (44–46).

Our ML model and SHAP analysis further identified advanced age as a prominent risk factor, with feature importance ranking second only to fibrinogen. Advanced age emerged as a strong predictor of lower extremity arterial embolism and thrombosis, likely due to its close association with the high prevalence of lower extremity atherosclerosis and cardiac arrhythmias (particularly atrial fibrillation). Epidemiological data indicate that the prevalence of peripheral artery disease increases continuously with advancing age (47). Aging itself is a key driver of atherosclerosis progression (48) and may further promote thrombosis through multiple pathways, including chronic inflammation (49), a hypercoagulable state (50), altered platelet function (51), and endothelial injury (52). Meanwhile, the prevalence of atrial fibrillation increases with age, significantly elevating the risk of cardioembolic events (53). Therefore, age not only drives *in situ* thrombosis by accelerating atherosclerosis but also exacerbates the burden of lower extremity arterial embolism by increasing the risk of cardioembolic events.

The XGBoost model developed in this study achieved an AUC of 0.951 in the test set. However, these high performance metrics must be interpreted with caution. Given the single-center, case-control design and the strong separation between the case and control groups, the reported AUC may reflect the optimism inherent in internal validation, potentially overestimating the model's real-world performance. The absence of external validation, temporal validation, or bootstrap optimism correction further underscores the need for prudence when applying these findings. Future multi-center prospective studies incorporating independent external validation cohorts are essential to rigorously evaluate the true predictive efficacy of this model in broader, more heterogeneous clinical populations. Similarly, the results of the RCS analysis should be interpreted with caution. Notably, this threshold (8.70 fl) falls below the standard reference interval (9.4–12.5 fl), suggesting that a markedly reduced MPV serves as a specific warning sign for high thrombotic risk. However, since MPV measurements vary across different hematology analyzers, this absolute cut-off may be assay- and center-specific. Therefore, it may not be universally applicable, and other institutions should recalibrate this threshold based on their local reference ranges before clinical implementation. Finally, although the proposed mechanisms provide a plausible explanation for the paradoxical association between low MPV and thrombotic risk, these biological interpretations remain largely speculative and require validation in large-scale prospective studies.

This study has several limitations. First, the retrospective design may introduce selection bias. Specifically, the exclusion of patients with missing key laboratory indicators not only reduced the sample size but also potentially introduced selection bias. Furthermore, arterial embolism and *in situ* arterial thrombosis were combined into a single endpoint due to the limited sample size, which precluded meaningful subgroup analyses despite their distinct pathophysiological mechanisms. Additionally, variable selection was primarily based on clinical prior knowledge and data availability, potentially omitting other influential factors. Second, the control group was not composed of completely healthy individuals, but rather patients with uncomplicated varicose veins. Although the majority of these controls did not have concurrent cardiovascular diseases, this choice inevitably introduced spectrum bias, meaning that the model's high performance may partly reflect these baseline disparities rather than its pure predictive efficacy in the general population. Third, the model demonstrated relatively low sensitivity, which limits its clinical utility as a standalone screening tool for this limb-threatening condition, and it lacked an independent external validation cohort, leaving its generalizability uncertain. Finally, the MR analysis has certain limitations. The evidence for MPV being protective was only marginally significant (*P* = 0.038) accompanied by significant heterogeneity (*P* < 0.001), which weakens the confidence in this specific causal interpretation. Furthermore, while the clinical data were derived from a Chinese population, the GWAS data used for causal inference were obtained from European populations, and future studies using Asian genomic data are needed to validate the cross-population applicability of the conclusions.

## Data Availability

The original contributions presented in the study are included in the article/Supplementary Material, further inquiries can be directed to the corresponding author/s.
