# Supplementary material for: Interpretable machine learning and mendelian randomization identify risk factors for lower extremity arterial embolism and thrombosis
Source: Front Cardiovasc Med. 2026 Jul 24;13:1827210. doi: 10.3389/fcvm.2026.1827210 (PMC13447431; doi:10.3389/fcvm.2026.1827210)
Supplement: Supplementary file 1 [file Datasheet1.docx]

**Table S1. GWAS dataset sources, populations, and download links**

| **Traits** | **Data sources** | **Population** | **Sample size** | **Data Download Links** |
| --- | --- | --- | --- | --- |
| Mean platelet volume | IEU | European | 460935 | https://opengwas.io/datasets/ebi-a-GCST90002346 |
| Aspartate aminotransferase levels | IEU | European | 436275 | https://opengwas.io/datasets/ebi-a-GCST90025980 |
| Gamma glutamyltransferase | IEU | European | 344104 | https://opengwas.io/datasets/ukb-d-30730_irnt |
| Serum urea levels | IEU | European | 437580 | https://opengwas.io/datasets/ebi-a-GCST90025991 |
| Ever smoked | IEU | European | 461066 | https://opengwas.io/datasets/ukb-b-20261 |
| Cerebrovascular disease | GWAS Catalog | mixed | 606181 | https://ftp.ebi.ac.uk/pub/databases/gwas/summary_statistics/GCST90480001-GCST90481000/GCST90480195/ |
| Diabetes mellitus | GWAS Catalog | European | 408959 | https://ftp.ebi.ac.uk/pub/databases/gwas/summary_statistics/GCST90435001-GCST90436000/GCST90435700/ |
| Estimated glomerular filtration rate (creatinine) | IEU | European | 1004040 | https://opengwas.io/datasets/ebi-a-GCST90103634 |
| Arterial embolism and thrombosis of lower extremity artery | finngen | European | 464403 | https://storage.googleapis.com/finngen-public-data-r12/summary_stats/release/finngen_R12_I9_ARTEMBTHRLOW.gz |

Table S2. Comparison of baseline characteristics between included patients and those excluded due to missing data

| **Characteristic** | **Overall**  N = 287^1^ | **Included**  N = 238^1^ | **Excluded due to missing data**  N = 49^1^ | **p-value**^2^ |
| --- | --- | --- | --- | --- |
| **Sex** |  |  |  | 0.424 |
| Female | 100 (35%) | 80 (34%) | 20 (41%) |  |
| Male | 187 (65%) | 158 (66%) | 29 (59%) |  |
| **year** | 76 (67, 83) | 76 (66, 83) | 77 (69, 83) | 0.510 |
| **DM** |  |  |  | 0.792 |
| NO | 218 (76%) | 182 (76%) | 36 (73%) |  |
| YES | 69 (24%) | 56 (24%) | 13 (27%) |  |
| **HBP** |  |  |  | 1.000 |
| NO | 150 (52%) | 124 (52%) | 26 (53%) |  |
| YES | 137 (48%) | 114 (48%) | 23 (47%) |  |
| **CD** |  |  |  | 0.133 |
| NO | 226 (79%) | 183 (77%) | 43 (88%) |  |
| YES | 61 (21%) | 55 (23%) | 6 (12%) |  |
| **CHD** |  |  |  | 0.087 |
| NO | 231 (81%) | 197 (83%) | 34 (71%) |  |
| YES | 55 (19%) | 41 (17%) | 14 (29%) |  |
| Missing | 1 | 0 | 1 |  |
| **Smoking** |  |  |  | 0.253 |
| NO | 185 (65%) | 150 (63%) | 35 (73%) |  |
| YES | 101 (35%) | 88 (37%) | 13 (27%) |  |
| Missing | 1 | 0 | 1 |  |
| **Alcohol consumption** |  |  |  | 0.890 |
| NO | 192 (68%) | 160 (67%) | 32 (70%) |  |
| YES | 92 (32%) | 78 (33%) | 14 (30%) |  |
| Missing | 3 | 0 | 3 |  |
| **TC** | 4.56 (3.81, 5.33) | 4.51 (3.80, 5.22) | 5.14 (3.94, 5.95) | 0.091 |
| Missing | 27 | 0 | 27 |  |
| **TG** | 1.29 (0.99, 1.95) | 1.27 (0.96, 1.89) | 1.42 (1.13, 2.56) | 0.140 |
| Missing | 27 | 0 | 27 |  |
| **HDL-C** | 1.29 (1.07, 1.63) | 1.28 (1.06, 1.63) | 1.44 (1.14, 1.63) | 0.578 |
| Missing | 27 | 0 | 27 |  |
| **LDL-C** | 2.70 (2.04, 3.34) | 2.68 (2.02, 3.27) | 3.16 (2.16, 3.76) | 0.089 |
| Missing | 27 | 0 | 27 |  |
| **ApoA1** | 1.40 (1.20, 1.63) | 1.39 (1.20, 1.63) | 1.59 (1.21, 1.77) | 0.112 |
| Missing | 30 | 0 | 30 |  |
| **ApoB** | 0.91 (0.72, 1.07) | 0.91 (0.72, 1.07) | 0.99 (0.75, 1.22) | 0.294 |
| Missing | 30 | 0 | 30 |  |
| **ALT** | 21.10 (14.15, 35.45) | 22.95 (14.70, 36.20) | 17.00 (12.40, 26.50) | 0.044 |
| Missing | 15 | 0 | 15 |  |
| **AST** | 26.20 (19.20, 42.15) | 26.35 (19.60, 45.30) | 22.00 (17.60, 32.10) | 0.054 |
| Missing | 15 | 0 | 15 |  |
| **TP** | 68.80 (64.15, 73.30) | 68.65 (64.10, 73.30) | 69.45 (64.50, 72.90) | 0.831 |
| Missing | 15 | 0 | 15 |  |
| **ALB** | 40.20 (37.70, 42.95) | 40.20 (37.60, 42.90) | 40.15 (38.30, 43.30) | 0.655 |
| Missing | 15 | 0 | 15 |  |
| **GGT** | 34.35 (21.80, 59.50) | 34.35 (22.70, 57.70) | 33.85 (20.30, 81.10) | 0.900 |
| Missing | 7 | 0 | 7 |  |
| **Urea** | 6.60 (5.23, 9.40) | 6.52 (5.19, 9.40) | 7.06 (6.15, 9.05) | 0.387 |
| **UA** | 340.10 (273.70, 426.40) | 339.60 (268.30, 427.00) | 354.10 (292.90, 411.20) | 0.725 |
| **Crea** | 75.70 (61.20, 93.10) | 75.85 (61.30, 92.70) | 71.70 (60.90, 96.80) | 0.634 |
| **GFR** | 82.10 (60.70, 92.90) | 82.15 (61.00, 92.70) | 80.20 (60.40, 95.00) | 0.881 |
| **PT** | 13.30 (12.60, 14.20) | 13.30 (12.50, 14.10) | 13.50 (12.80, 14.30) | 0.388 |
| **APTT** | 33.10 (29.60, 37.80) | 32.90 (29.10, 37.80) | 34.50 (31.30, 37.80) | 0.076 |
| **TT** | 17.30 (16.30, 18.70) | 17.40 (16.30, 18.80) | 16.90 (15.80, 17.90) | 0.036 |
| **Fib** | 3.92 (3.19, 5.15) | 3.86 (3.12, 5.04) | 4.22 (3.31, 5.57) | 0.177 |
| **MPV** | 9.80 (9.00, 10.90) | 9.80 (9.00, 10.90) | 9.70 (8.90, 10.90) | 0.473 |
| Missing | 4 | 0 | 4 |  |
| **PLT** | 202.00 (150.00, 272.00) | 199.00 (150.00, 271.00) | 218.00 (166.00, 295.00) | 0.497 |
| Missing | 4 | 0 | 4 |  |
| ^1^n (%); mean±SD or median (Q1, Q3) | | | | |
| ^2^Continuous variables: normally distributed data are presented as mean±SD and compared using Student’s t test; non-normally distributed data are presented as median (IQR) and compared using the Mann–Whitney U test. Categorical variables were compared using the chi-square test. | | | | |
| DM: Diabetes Mellitus; HBP: Hypertension; CD: Cerebrovascular Disease; CHD: Coronary Heart Disease; TC: Total Cholesterol; TG: Triglycerides; HDL-C: High-Density Lipoprotein Cholesterol; LDL-C: Low-Density Lipoprotein Cholesterol; ApoA1: Apolipoprotein A1; ApoB: Apolipoprotein B; ALT: Alanine Aminotransferase; AST: Aspartate Aminotransferase; TP: Total Protein; ALB: Albumin; GGT: Gamma-Glutamyl Transferase; UA: Uric Acid; Crea: Creatinine; GFR: Glomerular Filtration Rate; PT: Prothrombin Time; APTT: Activated Partial Thromboplastin Time; TT: Thrombin Time; Fib: Fibrinogen; MPV: Mean Platelet Volume; PLT: Platelet Count. | | | | |

**Table S3. Mendelian randomization results**

| exposure | method | nsnp | β | OR | 95%CI | *P* | *P*_Heterogeneity_ | *P* _pleiotropy_ |  |
| --- | --- | --- | --- | --- | --- | --- | --- | --- | --- |
| CD | MR Egger | 18 | 1.184 | 3.269 | 0.537, 19.913 | 0.217 | 0.136 |  |  |
| CD | Weighted median | 18 | 0.565 | 1.76 | 0.923, 3.356 | 0.086 |  |  |  |
| CD | Inverse variance weighted | 18 | 0.573 | 1.773 | 1.043, 3.015 | 0.035 | 0.152 | 0.497 |  |
| GGT | MR Egger | 249 | 0.115 | 1.122 | 0.834 ,1.508 | 0.449 | 0.204 |  |  |
| GGT | Weighted median | 249 | 0.365 | 1.441 | 1.048, 1.981 | 0.025 |  |  |  |
| GGT | Inverse variance weighted | 249 | 0.283 | 1.327 | 1.105, 1.595 | 0.003 | 0.191 | 0.157 |  |
|  |  |  |  |  |  |  |  |  |  |
| MPV | MR Egger | 489 | -0.121 | 0.886 | 0.743, 1.056 | 0.176 | 8.01 × 10⁻⁴ |  |  |
| MPV | Weighted median | 489 | -0.147 | 0.863 | 0.696, 1.071 | 0.181 |  |  |  |
| MPV | Inverse variance weighted | 489 | -0.129 | 0.879 | 0.778, 0.993 | 0.038 | 8.89 × 10⁻⁴ | 0.905 |  |
| AST levels | MR Egger | 261 | -0.067 | 0.935 | 0.612, 1.429 | 0.758 | 0.264 |  |  |
| AST levels | Weighted median | 261 | -0.131 | 0.877 | 0.578, 1.331 | 0.538 |  |  |  |
| AST levels | Inverse variance weighted | 261 | 0.055 | 1.057 | 0.835, 1.338 | 0.645 | 0.272 | 0.497 |  |
| Serum urea levels | MR Egger | 186 | -0.075 | 0.927 | 0.495, 1.738 | 0.814 | 0.198 |  |  |
| Serum urea levels | Weighted median | 186 | -0.074 | 0.929 | 0.567, 1.521 | 0.77 |  |  |  |
| Serum urea levels | Inverse variance weighted | 186 | -0.074 | 0.928 | 0.690, 1.249 | 0.624 | 0.212 | 0.997 |  |
| Ever smoked | MR Egger | 16 | -0.932 | 0.394 | 7.12 × 10⁻⁶ , 3.29 × 10⁸ | 0.892 | 0.038 |  |  |
| Ever smoked | Weighted median | 16 | 3.068 | 21.49 | 0.588, 785.006 | 0.095 |  |  |  |
| Ever smoked | Inverse variance weighted | 16 | 2.321 | 10.182 | 0.472, 219.830 | 0.139 | 0.049 | 0.625 |  |
| DM | MR Egger | 36 | -0.143 | 0.867 | 0.625 , 1.203 | 0.399 | 0.937 |  |  |
| DM | Weighted median | 36 | -0.04 | 0.961 | 0.776 , 1.190 | 0.714 |  |  |  |
| DM | Inverse variance weighted | 36 | 0.042 | 1.043 | 0.905 , 1.202 | 0.563 | 0.923 | 0.228 |  |
| Estimated glomerular filtration rate (creatinine) | MR Egger | 18 | 3.88 | 48.412 | 7.12 × 10⁻⁶ , 3.29 × 10⁸ | 0.635 | 0.332 |  |  |
| Estimated glomerular filtration rate (creatinine) | Weighted median | 18 | 3.16 | 23.567 | 0.252, 2205.318 | 0.172 |  |  |  |
| Estimated glomerular filtration rate (creatinine) | Inverse variance weighted | 18 | 2.287 | 9.843 | 0.382 , 253.321 | 0.168 | 0.395 | 0.842 |  |

CD, cerebrovascular disease; GGT, gamma-glutamyltransferase; MPV, mean platelet volume; AST, aspartate aminotransferase; DM, diabetes mellitus; Nsnp: Number of single nucleotide polymorphisms; *P*Heterogeneity, P value for heterogeneity test (*P* < 0.05 indicates significant heterogeneity); *P*pleiotropy, P value for horizontal pleiotropy test (*P* < 0.05 suggests presence of horizontal pleiotropy).

Figure S1. Flowchart of patient selection and cohort assembly. The flowchart shows the screening, exclusions (side boxes), and final inclusion of participants in the case and control groups.

Figure S2. Restricted cubic spline (RCS) analysis of mean platelet volume (MPV) and the risk of lower extremity arterial embolism and thrombosis after adjustment for confounders. The RCS curve shows the association between MPV and thrombotic risk across the MPV range after adjustment for confounders and allows assessment of nonlinearity.

Figure S3. Web-based interface of the lower extremity arterial embolism and thrombosis prediction tool. Web-based interface of the prediction tool, including the input panel, predicted risk output, and variable-importance display for model interpretability.
